# Primary peritonitis: an enigmatic case and literature exploration-diagnostic challenges and therapeutic approaches (case report)

**DOI:** 10.11604/pamj.2025.50.23.43398

**Published:** 2025-01-13

**Authors:** Amina Chaka, Wael Boujelbène, Amin Chaabouni, Amin Zouari, Mahmoud Kammoun, Ines Ben Hammouda, Housem Harbi, Salah Boujelbène

**Affiliations:** 1 General Surgery Department, Habib Bourguiba Hospital, Sfax, Tunisia

**Keywords:** Primary peritonitis, laparoscopy, antibiotics, case report

## Abstract

Primary peritonitis, also known as spontaneous peritonitis, is rare and has no obvious intra-abdominal origin. Antibiotic therapy is usually sufficient. However, surgery is sometimes necessary when the primary character of the peritonitis is uncertain. We report herein the case of a 24-year-old patient, with no particular medical history and who presented to the emergency department for diffuse abdominal pain with fever. Abdominal examination found diffuse abdominal tenderness with hyperleukocytosis. A computed tomography scan showed generalized intraperitoneal effusion with no obvious abdominal infectious hotbed, nor pneumoperitoneum. However, it showed lateral basal pneumonia. Laparoscopic exploration confirmed the diagnosis of peritonitis but didn´t find any obvious cause. She had a peritoneal cleansing with an appendectomy of principle. Then she was put on probabilistic broad-spectrum parenteral antibiotic therapy. However bacteriological examination of the peritoneal fluid and blood cultures were negative. The surgical postoperative course was uneventful and she was discharged at post-operative day 5.

## Introduction

Primary or spontaneous peritonitis is defined as an infectious process involving the peritoneal cavity and originating neither from an inflammatory process of the peritoneal cavity, nor from a visceral perforation, nor a penetrating abdominal wound [1]. Excluding infections of ascites fluid in patients with hepatic cirrhosis and peritonitis complicating peritoneal dialysis, this entity remains very rare with a prevalence <1% of all peritonitis [1-4]. Although the treatment is often medical with antibiotics, surgery is sometimes necessary when the primary character of the peritonitis is uncertain and in the absence of a suggestive clinical context (cirrhosis, peritoneal dialysis, nephrotic syndrome). Our objective was to describe the diagnostic difficulties of the primary character of peritonitis in a 24-year-old patient and to conduct a recent literature review of this rare entity to adopt an adequate therapeutic approach.

## Patient and observation

**Patient information:** a 24-year-old woman, without any particular medical history, presented to the emergency department for acute abdominal pain for 3 days with fever and vomiting. In addition, she reported a dry cough that started 10 days ago.

**Clinical findings:** her clinical examination showed a fever of 38.7°C, diffuse abdominal tenderness, and maximum pain in the hypogastric region. There was no hemodynamic failure (blood pressure at 130/85 mmHg and heart rate at 85 beats per minute). Besides, she had no leucorrhea.

**Figure 1:**
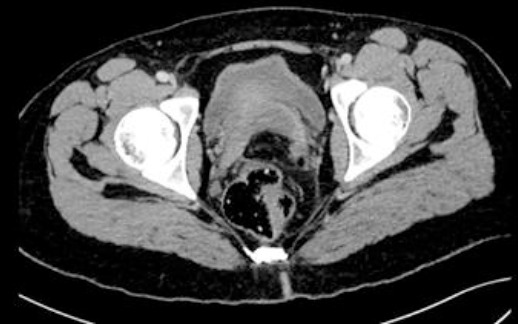
intraperitoneal fluid and thick pelvic peritoneal sheets

**Figure 2:**
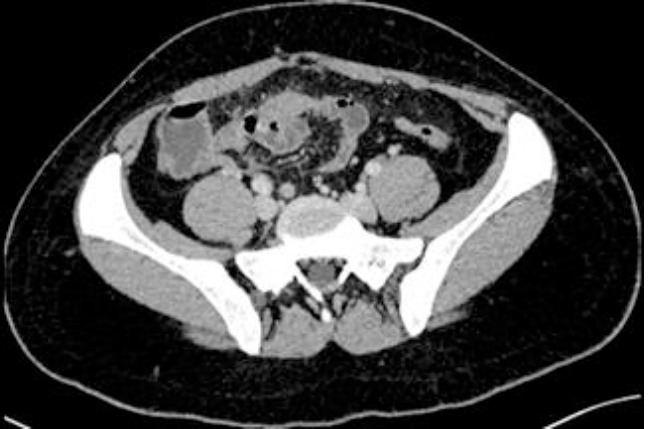
normal appearance of the appendix in a computed tomography scan

**Diagnostic assessment:** blood tests showed a biological inflammatory syndrome with hyperleukocytosis (15200 elements/mm^3^ and high CRP level at 65 mg/l). Lipasemia and beta-HCG levels were normal. Thoraco-abdominal CT scan showed free intraperitoneal fluid of medium abundance and a thickening of the pelvic peritoneal sheets, but there was no pneumoperitoneum (Figure 1). It also showed few inflammatory lymph nodes. The appendix was not swollen, and the ovaries were multi-follicular (Figure 2). CT-thoracic views showed bilateral poster basal parenchymal condensations with an aerated bronchogram consistent with bilateral basal pneumonia.

**Figure 3:**
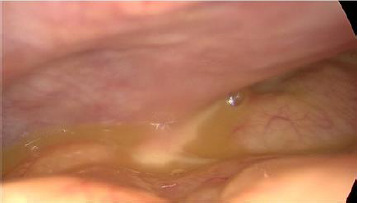
intraperitoneal purulent effusion

**Figure 4:**
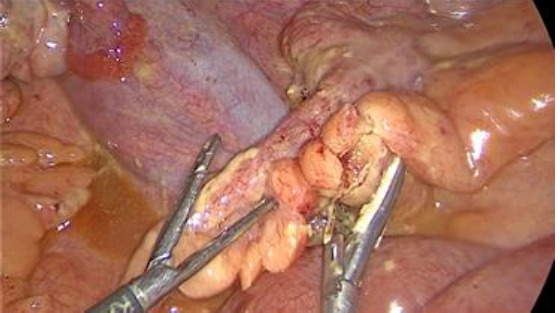
per operative exploration: normal aspect of the appendix

**Figure 5:**
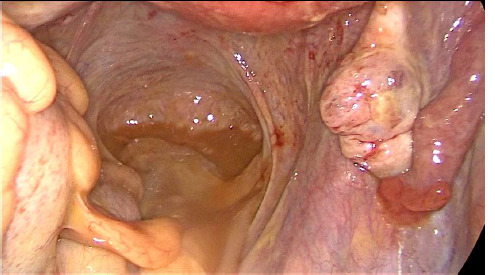
normal aspect of the ovaries and fallopian tubes

**Therapeutic intervention:** then, the patient underwent an emergent laparoscopy. There was a large purulent effusion with false membranes (Figure 3). The appendix, the ovaries, the fallopian tubes, and the gallbladder were normal (Figure 4, Figure 5). Besides, there was no perforation in the hollow organs. A bacteriological sample was taken, and a peritoneal cleansing and an appendectomy of principle were performed. Simultaneously, a broad-spectrum probabilistic parenteral antibiotic therapy was started, combining ceftriaxone, metronidazole, and levofloxacin. The bacteriological examination of the peritoneal fluid and blood cultures were negative.

**Follow-up and outcomes:** the postoperative course was uneventful and the patient was discharged on postoperative day 5 with additional oral antibiotic therapy for 5 days.

**Patient perspective:** the patient was satisfied with treatment with good follow-up after one year.

**Informed consent:** the patient gave her full consent to publish her case.

## Discussion

Primary peritonitis is usually reported in patients with cirrhosis, ascites, nephrotic syndrome, or lupus [1-4]. Apart from these risk factors, it remains infrequent, accounting for approximately 1% of all peritonitis [1-3,5]. Patients with primary peritonitis, typically present with generalized abdominal pain, vomiting, and fever [3,6]. It may rapidly progress to multi-visceral failure if it isn´t treated in time [2,3,5-7]. Clinical examination should look for a possible ORL, pulmonary, or gynecological infection that would have caused peritoneal contamination in a hematogenous way [1-3,6,8]. More rarely, the lymphatic way, the trans diaphragmatic contamination, or intestinal bacteria translocation are incriminated [1,7,8]. However, the infectious investigation may sometimes be negative [1,8].

The bacterial inoculation is often mono-microbial. The Pneumococcus and the group A beta-hemolytic streptococcus are the main bacteria involved in primary peritonitis [2,6,8]. Yamou and al reported two cases of primary peritonitis related to Escherichia Coli and a gram-negative bacillus that could not be specified [1]. The causal germ couldn´t also be specified in our case, but the primitive peritonitis was related to the pneumonia. The abdominal Ct-scan should show a generalized intra-abdominal effusion with thickening of the peritoneal layers but without pneumoperitoneum and especially without any obvious intra-abdominal infectious cause [3,5,7].

It is worth noting that the diagnosis of primary peritonitis is usually done retrospectively with a suggestive clinical context while all other causes of peritonitis have been ruled out by imaging and mainly by surgical exploration [1,3]. Thus, Yamou R *et al*. suggested that surgical exploration is mandatory in primary peritonitis [1]. In a 2017 literature review, among 46 cases of primary group A streptococcal peritonitis, 38 patients underwent surgery (28 had laparotomy and 10 had laparoscopy) [3]. Peritoneal cleansing, either by laparotomy or laparoscopy, is usually performed and remains very efficient [1]. In some cases, antibiotic therapy alone may be sufficient, particularly in the case of a clinical context suggestive of liver cirrhosis or peritoneal dialysis [3]. But aside from these situations, antibiotic therapy for primary peritonitis remains not consensual [3].

**Figure 6:**
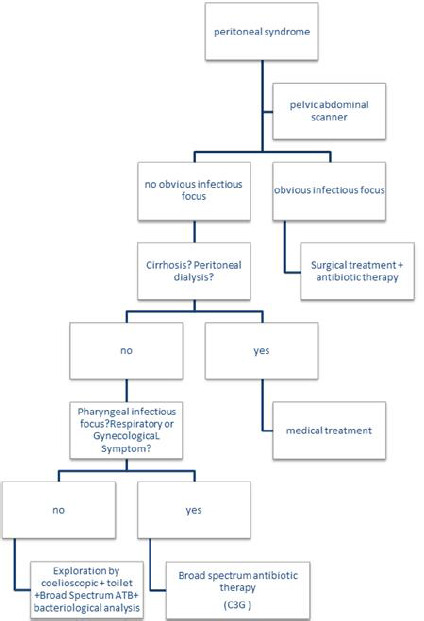
management of primary peritonitis

In practice, it is initially probabilistic then it should be adapted to the antibiogram [3,9,10]. Primary peritonitis remains a serious disease whose prognosis depends mainly on the patient´s medical history, the patient's condition at diagnosis, the causal germ, and the treatment timeframe. Due to the limited number of cases in the literature and the unavailability of a standardized treatment approach, we propose this approach to the management of primary peritonitis (Figure 6).

## Conclusion

Although, primary peritonitis is rare, early diagnosis and emergent management are crucial to avoid multi-visceral failure and fatal outcomes. Peritoneal cleansing and antibiotic therapy are the mainstays of treatment. An infectious investigation searching for the primary septic focus is necessary to adapt the antibiotic therapy. However, the causative germ may sometimes still be unknown.
